# Mangiferin Attenuates Diabetic Nephropathy by Inhibiting Oxidative Stress Mediated Signaling Cascade, TNFα Related and Mitochondrial Dependent Apoptotic Pathways in Streptozotocin-Induced Diabetic Rats

**DOI:** 10.1371/journal.pone.0107220

**Published:** 2014-09-18

**Authors:** Pabitra Bikash Pal, Krishnendu Sinha, Parames C. Sil

**Affiliations:** Division of Molecular Medicine, Bose Institute, Kolkata, India; IISER-TVM, India

## Abstract

Oxidative stress plays a crucial role in the progression of diabetic nephropathy in hyperglycemic conditions. It has already been reported that mangiferin, a natural C-glucosyl xanthone and polyhydroxy polyphenol compound protects kidneys from diabetic nephropathy. However, little is known about the mechanism of its beneficial action in this pathophysiology. The present study, therefore, examines the detailed mechanism of the beneficial action of mangiferin on STZ-induced diabetic nephropathy in Wister rats as the working model. A significant increase in plasma glucose level, kidney to body weight ratio, glomerular hypertrophy and hydropic changes as well as enhanced nephrotoxicity related markers (BUN, plasma creatinine, uric acid and urinary albumin) were observed in the experimental animals. Furthermore, increased oxidative stress related parameters, increased ROS production and decreased the intracellular antioxidant defenses were detected in the kidney. Studies on the oxidative stress mediated signaling cascades in diabetic nephropathy demonstrated that PKC isoforms (PKCα, PKCβ and PKCε), MAPKs (p38, JNK and ERK1/2), transcription factor (NF-κB) and TGF-β1 pathways were involved in this pathophysiology. Besides, TNFα was released in this hyperglycemic condition, which in turn activated caspase 8, cleaved Bid to tBid and finally the mitochorndia-dependent apoptotic pathway. In addition, oxidative stress also disturbed the proapoptotic-antiapoptotic (Bax and Bcl-2) balance and activated mitochorndia-dependent apoptosis via caspase 9, caspase 3 and PARP cleavage. Mangiferin treatment, post to hyperglycemia, successfully inhibited all of these changes and protected the cells from apoptotic death.

## Introduction

Diabetic mellitus is one of the most recognizable endocrine metabolic disorders fundamentally characterized by hyperglycemia via disruption of carbohydrate, fat and protein metabolism from insufficiency of secretion or action of endogenous insulin (WHO). Hyperglycemia is a well distinguished pathogenic factor of chronic complications in diabetic mellitus and not only generates excessive free radicals (reactive oxygen species; ROS) but also attenuates antioxidative machineries through glycation of the antioxidant enzymes [1]. Streptozotocin (STZ) is commonly used as a diabetic inducer in experimental animals and its toxicity is generated by nitric oxide (NO) on pancreatic β-cells. The cellular toxicity of STZ is linked with the ROS formation resulting oxidative damage of various organ tissues [2]. Hence, oxidative stress has been considered to be a general pathogenic factor of diabetic complications including nephropathy [3]. Diabetic nephropathy is the most serious micro vascular complication of diabetes mellitus and the most common cause of the end stage renal disease (ESRD). This is usually found in both type 1 or type 2 diabetes worldwide [4]. It is caused by the damage to small blood vessels in the kidneys that in turn become less efficient or ultimately fail to function [5]. Diabetic nephropathy has been characterized by glomerular hypertrophy, glomerular hyperfiltration, increased urinary albumin secretion, increased basement membrane thickness and mesangial expansion with the accumulation of extracellular matrix proteins (ECM) [6], [7].

Hyperglycemia is strongly associated with increased production of reactive oxygen species (ROS). The plausible major sources of ROS in the diabetic nephropathy are: the activation of polyol pathways, advanced glycation end products (AGEs), autoxidation of glucose, xanthine oxidase activity, mitochondrial respiratory chain deficiencies, NAD(P)H oxidase and nitric oxide synthase (NOS) [8], [3]. Therefore, a molecule possessing both hypoglycemic and antioxidant properties might be considered a protective agent against diabetic nephropathy [9], [10].

Herbal medicinal plants are of importance to the health of the individual as well as for the communities. The beneficial effects of these medicinal plants are typically due to the occurrence of several chemically active materials that generate a specific physiological action in the human body. Numerous herbal medicinal plants like *Terminalia arjuna* [11]–[14], *Phyllanthus niruri* [15]–[19], *Pithecellobium dulce* [20], *Cajanus indicus* [21]–[25], etc. are natural sources of antioxidants in India and worldwide. These bioactive antioxidants have been used for the treatment of various organ dysfunctions. In addition, fruits, vegetables, tea and wine are the major sources of flavonoids, xanthanoids or polyphenolic compounds and are typically well known to have strong antioxidant activity [26]. Mangiferin (1,3,6,7-Tetrahydroxyxanthone C2-β-D-glucoside; C_19_H_18_O_11_; M.W.- 422,34 g/mol), a natural C-glucosyl xanthone and polyhydroxy polyphenol compound, can be found in various plant species, such as the bark of mango tree (*Mangifera indica*) [27], [28]. Various earlier reports showed that mangiferin contains antidiabetic [29], antidiarrhea [30], anticancer [31], antiallergic [32], anti-HIV [33] antibacterial [34] and antioxidant [35] properties. In our previous studies we have also established that mangiferin act as a very good protective agent against Pb(NO_3_)_2_ induced oxidative stress in hepatic and cardiac pathophysiology by enhancing antioxidant defense and acting through apoptotic pathways [36], [37]. A number of literature reported the beneficial role of mangiferin in STZ induced type 1 diabetic nephropathy via oxidative stress, but the details of the mechanisms is still unknown.

In the present study we have, therefore, designed experiments to investigate the mechanisms underlying the protective action of mangiferin in renal oxidative damage induced by streptozotocin (STZ) type1 (insulin dependent) diabetes using rats as the working model.

## Materials and Methods

### Chemicals

STZ (Streptozotocin), bovine serum albumin (BSA), DHE (dihydroethidium) and Bradford reagent were purchased from Sigma-Aldrich Chemical Company (St. Louis, MO, USA). Antibodies were purchased from cell signaling technology, Inc. (CST) and Abcam. Kits for measurement of blood glucose, blood uria nitrogen (BUN), creatinine, uric acids were purchased from Span Diagnostic Ltd. India and kit for urinary albumin was purchased from Abcam. All other chemicals were bought from Sisco Research Laboratory, India.

### Animals

Adult male Wister rats weighing approximately 160–180 g were purchased from M/S Gosh Enterprises, Kolkata, India. Animals were acclimatized under laboratory conditions for 2 weeks prior to experiments. They were maintained under standard conditions of temperature (23±2°C) and humidity (50±10%) with an alternating 12 h light/dark cycles. The animals had free access to tap water and were fed a standard pellet diet (Agro Corporation Private Ltd., Bangalore, India). All the experiments with animals were carried out according to the guidelines of the Institutional Animal Ethical Committee (IAEC), Bose Institute, Kolkata (the permit number is IAEC/BI/3(I) cert./2010) and full details of the study was approved by both the IAEC and Committee for the Purpose of Control and Supervision on Experiments on Animals (CPCSEA), Ministry of Environment & Forests, New Delhi, India (the permit number is 95/99/CPCSEA).

### Extraction and isolation of mangiferin

Mangiferin was extracted in our laboratory following the method as described by Ghosh et al. [38]. Briefly, crudely powdered bark of *Mangifera indica* was thoroughly extracted thoroughly with ethanol (95%) in a Soxhlet apparatus for 56 h. The combined alcohol extracts was concentrated under reduced pressure until a yellow amorphous powder was obtained. The dried alcoholic extract was adsorbed on silica gel (60–120 mesh) and chromatographed in silica gel column packed in petroleum ether (60–80°). The column was eluted with chloroform: methanol (1∶1) which yielded mangiferin as a pale yellow amorphous powder that crystallized with the ethanol to form pale yellow needle shaped mangiferin crystals. Purity of the product has been checked by the HPLC, HRMS (ESI) analysis and NMR (^1^H, ^13^C) spectroscopy (data not shown).

### Determination of dose dependent activity of mangiferin by BUN level

For this study, rats were randomly distributed into seven groups each consisting of six animals. First two groups were served as normal control (received only water as vehicle) and STZ-treated (STZ single dose, 65 mg/kg mg/kg body weight, inject intraperitonally) respectively. Remaining five groups of animals were treated with five different doses of mangiferin (10 mg, 20 mg, 40 mg, 60 mg and 80 mg/kg body weight for 30 days, orally) after STZ administration.

### *In vivo* experimental design

Group 1 (Normal Group: abbreviated as Cont): Rats received neither STZ nor mangiferin, but received vehicle only.
Group 2 (Mangiferin treated Group: abbreviated as Mang): Rats received only an oral sample of mangiferin at a dose of 40 mg/kg body weight.
Group 3 (Diabetic Group: abbreviated as STZ): Overnight fasted rats received a single dose of streptozotocin (STZ, 65 mg/kg mg/kg body weight, inject intraperitonally) in 0.1 M cold citrate buffer (pH 4.5). The blood glucose levels above 250 mg/dl on the 3^rd^ day after STZ injection were considered as diabetic.
Group 4 (STZ+Mangiferin: abbreviated as STZ+Mang): Post treatment Group: Rats orally received mangiferin at a dose of 40 mg/kg body weight/day in water for 30 days orally from the 4^th^ day after STZ injection.

### Collection of blood, urine and kidney from experimental animals

The animals were sacrificed [anesthetizing with ketamin (IM) and thiobutabarbital (IP) at a dose of 30 and 50 mg/kg body wt respectively] after the experimental period and the kidneys were collected and stored at −80°C until further analysis. The body weight and kidney weight were measured and evaluated between groups. Blood samples were withdrawn from the caudal vena cava, collected in test tubes having heparin solution and centrifuged at 1,500 g for 10 min to obtain plasma. The plasma was instantly stored at −80°C until use. For albumin measurements urine was collected from the bladder and immediately stored at −80°C.

### Preparation of mitochondrial, cytosolic and microsomal fractions

The kidneys were minced, rinsed with PBS and homogenized in a Dounce glass homogenizer in homogenizing buffer (50 mM phosphate buffer, pH 7.5, containing 1 mM EDTA, 1.5 mM MgCl_2_, 10 mM KCl and supplemented with protease and phosphatase inhibitors). The homogenates were centrifuged for 10 min at 2,000 g at 4°C. The pellet was thrown away and the supernatant was re-centrifuged at 12,000 g for 10 min at 4°C. The pellet was then re-suspended in 200 mM mannitol, 50 mM sucrose, 10 mmol/L Hepes–KOH (pH 7.4) and stored as a mitochondrial fraction at −80°C. The final supernatant was further centrifuged at 105,000 g for 60 min at 4°C. The resulting microsomal pellets were then suspended in a 0.25 mM sucrose solution containing 1 mM EDTA and stored at −80°C until use. The supernatant was received and used as cytosolic fraction and stored at 4°C.

### Measurement of protein content

The protein content of the experimental samples was measured by the method of Bradford (1976) [39] using crystalline BSA as standard.

### Measurement of plasma glucose levels and kidney dysfunction markers

Plasma glucose levels and specific markers related to kidney dysfunction such as BUN, creatinine, uric acid in the plasma and urinary albumin were estimated using standard kits.

### Histological studies

For histological assessments, small segments of kidneys from the normal and experimental rats were fixed in 10% buffered formalin and were processed for paraffin sectioning. Sections of about 5 µm width were stained with hematoxylin and eosin (H&E) for assessment under light microscope.

### Measurement of lipid peroxidation and protein carbonyl content

The lipid peroxidation in terms of malondialdehyde (MDA) formation in kidney tissue homogenate (containing 1 mg of protein) was measured following the method of Esterbauer and Cheeseman [40].

Protein carbonyl contents were measured according to the methods Uchida and Stadtman [41].

### Measurement of intracellular ROS production

Intracellular ROS production was measured by using 2,7-dichlorofluorescein diacetate (DCFDA) as a probe according to the method of LeBel and Bondy [42] followed by some modifications introduced by Kim et al. [43]. The formation of DCF was assessed in a fluorescence spectrometer (HITACHI, Model No F4500) equipped with a FITC filter at the excitation wavelength of 488 nm and emission wavelength of 510 nm for 10 minutes.

The oxidative fluorescent dye dihydroethidium (DHE) was used to detect superoxide (O_2_^.**−**^) production in kidney from normal and experimental rats [44]. Cryosections (10 µm) from kidney tissue, were stained with the dye DHE (10 µmol/L) in a light-protected and humidified chamber for 30 min at 37°C. Images for each section were analyzed with a fluorescent microscope.

### Measurement of the activity of antioxidant enzymes

Activities of antioxidant enzymes such as superoxide dismutase (SOD), catalase (CAT), glutathione peroxidase (GP_X_) and glutathione reductase (GR) in the kidney tissue were measured following the methods as described elsewhere [45].

### Measurement of the levels of cellular metabolites

Cellular GSH levels were measured by using Ellman’s reagent (DTNB; 5,5-dithiobis-2-nitrobenzoic acid) [46]. Oxidized glutathione (GSSG) contents in the samples were measured following the method of Hissin and Hilf [47].

### Measurement of plasma AGEs level by ELISA

The levels of plasma AGEs was measured by ELISA kits according to the manufacturer’s instructions (Abcam, UK).

### Measurement of xanthine oxidase activity

The activity of xanthine oxidase was assessed by measuring the enzymatic oxidation of xanthine. The reactive mixture contained 1.9 mL of 50 mM potassium phosphate buffer, pH 7.5 and 1 mL of 0.15 mM xanthine. The reaction was started by adding 100 µL of kidney tissue extract and the increase in absorbance was measured at 290 nm for 4 min.

### Measurement of renal hydroxyproline level

The kidney hydroxyproline levels were measured according to the method of Woessner (1961) [48].

### Determination of mitochondrial membrane potential

The mitochondrial membrane potential from isolated mitochondrial fraction of kidney tissue was carried out by using a FACScan flow cytometer with an argon laser excitation at 488 nm and a 525 nm band-pass filter. Mitochondrial membrane potential (Δ*Ψ* ***_m_***) has been estimated on the basis of cell preservation of the fluorescent cationic probe rhodamine 123.

### Agarose gel electrophoresis for DNA fragmentation

The DNA fragmentation assay was performed by using electrophoresing genomic DNA samples, isolated from normal as well as experimental kidney, on agarose/EtBr gel by the procedure described by Sellins and Cohen [49].

### TUNEL assay for DNA fragmentation

Paraffin embedded renal tissue sections (5 µm) was warmed for 30 min (64°C), deparaffinized and rehydrated. Terminal transferase mediated dUTP nick end-labeling of nuclei has been performed by using APO-BrdU TUNEL Assay kit (A-23210; Molecular Probes, Eugene, OR) following the manufacturer’s protocol.

### Immunoblotting

An equal amount of protein (50 µg) from each sample was resolved by 10% SDS-PAGE and transferred to PVDF membrane. Membranes were blocked at room temperature for 2 h in blocking buffer containing 5% non-fat dry milk to prevent non-specific binding. The membranes were then incubated with each of these anti-PKCα (1∶250 dilution), anti-PKCβ (1∶250 dilution), anti-PKCε (1∶250 dilution), anti-ERK (1∶1,000 dilution), anti-JNK (1∶1,000 dilution), anti-p38 (1∶1,000 dilution), anti-NF-κB(1∶1,000 dilution), anti-TGF-β1(1∶1,000 dilution), anti-TNFα (1∶1,000 dilution), anti-caspase-8 (1∶1,000 dilution), anti-t-Bid (1∶1,000 dilution), anti-Bid (1∶1,000 dilution), anti-Bcl-2 (1∶1,000 dilution), anti-Bax (1∶1,000 dilution), anti-cytochrome C (1∶1,000 dilution), anti-caspase-3 (1∶1,000 dilution), anti-caspase-9 (1∶1,000 dilution) and anti-PARP (1∶1000) primary antibodies separately at 4°C overnight. The membranes were washed in TBST (50 mmol/L Tris–HCl, pH 7.6, 150 mmol/L NaCl, 0.1% Tween 20) for 30 min and incubated with appropriate HRP-conjugated secondary antibody (1∶2,000 dilution) for 2 h at room temperature and developed by the HRP substrate, 3,30 diaminobenzidine tetrahydrochloride (DAB) system (Bangalore Genei, India).

### Statistical analysis

All the experimental values were expressed as mean ± SEM (n = 6). Significant differences between the groups were determined with SPSS 10.0 software (SPSS Inc., Chicago, IL, USA) for Windows using one-way analysis of variance (ANOVA) and the group means were compared by Duncan’s Multiple Range Test (DMRT). A difference was considered significant at the p<0.05 level.

## Results

### Dose dependent effect of mangiferin by BUN level

**Figure 1:**
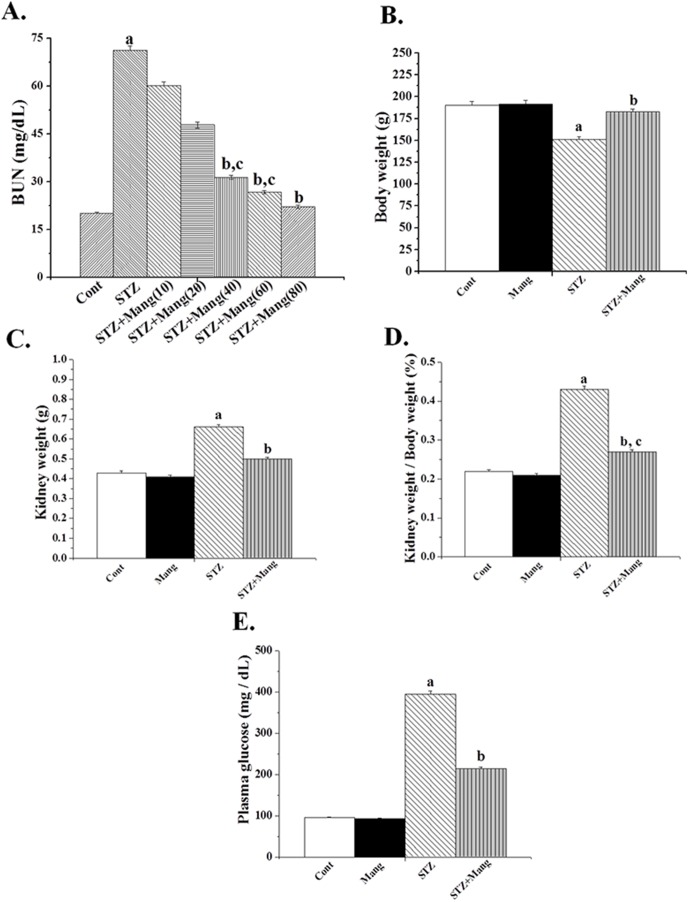
Effects of mangiferin (Mang) on the body weight, kidney weight, plasma glucose and nephrotoxicity of STZ-induced type 1 diabetic rats. Cont: normal control, Mang: treated with mangiferin, STZ: STZ-induced (diabetic), STZ+Mang: Mangiferin treated post to STZ-induced. (A) Dose dependent effect of mangiferin on BUN level against STZ induced toxicity in the kidney tissue of the experimental rats. [Cont: BUN level in normal mice, STZ: BUN level in STZ induced mice, STZ+Mang(10), STZ+Mang(20), STZ+Mang(40), STZ+Mang(60) and STZ+Mang(80): BUN level in mangiferin (Mang) treated mice for 30 days at a dose of 10, 20, 40, 60 and 80 mg/kg body weight respectively post to STZ administration], (B) Body weight, (C) kidney weight, (D) kidney weight to body weight ratio, (E) plasma glucose. Each column represents mean ± SEM, n = 6. “a” indicates the significant difference between the normal control and STZ-induced groups, “b” indicates the significant difference between the STZ-induced and mangiferin treated groups and “c” indicates the significant difference between the STZ+Mangiferin group and normal control group (P^a^<0.05, P^b^<0.05, P^c^<0.05).

BUN assay was used to determine the optimum dose of mangiferin for its protective action of kidney tissue against STZ-induced oxidative damage. Experimental results suggest that STZ-induced increased BUN level and that could be prevented by mangiferin treatment linearly up to a dose of 40 mg/kg body weight for 30 days (Figure 1A). This dose of mangiferin has, therefore, been used for the subsequent experiments.

### Effects of mangiferin on the body weight, kidney weight and plasma glucose of STZ induced diabetic rats

Approximately 90% of rats injected with STZ developed type 1 diabetes characterized by the significant increase in plasma glucose level (395 mg/dL) (Figure 1E). Physical inertia was detected in the STZ-induced diabetic rats; animals gained kidney weight and the kidney to body weight ratio (a marker for the development of diabetic nephropathy) was also increased (Figure 1B, C, D) compared to normal rats. Post-treatment with mangiferin for 30 days after STZ exposure, however, decreased the plasma glucose levels (214 mg/dL) and reduced the growth accelerating activities suggesting its role as a good anti-hyperglycemic and growth-inhibiting agent in STZ induced diabetic animals.

### Effects of mangiferin on STZ-induced nephrotoxicity

**Figure 2:**
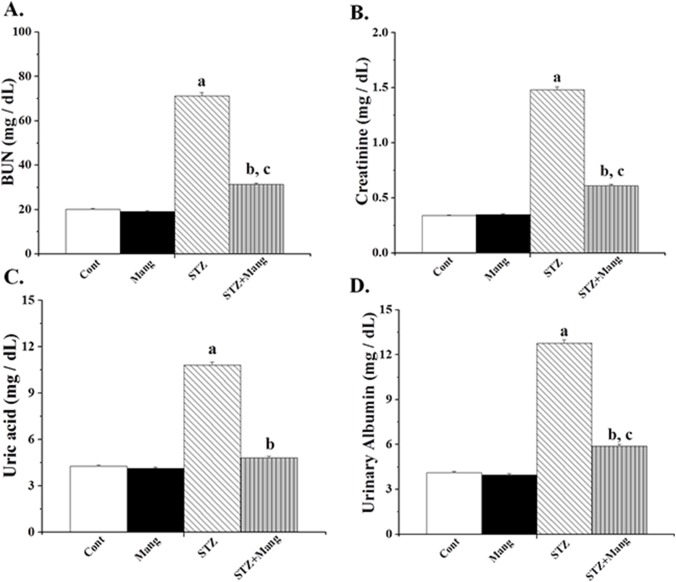
Effects of mangiferin (Mang) on the nephrotoxicity of STZ-induced type 1 diabetic rats. Cont: normal control, Mang: treated with mangiferin, STZ: STZ-induced (diabetic), STZ+Mang: Mangiferin treated post to STZ-induced. (A) BUN, (B) Creatinine, (C) Uric acid and (D) urinary albumin were measured. Each column represents mean ± SEM, n = 6. “a” indicates the significant difference between the normal control and STZ-induced groups, “b” indicates the significant difference between the STZ-induced and mangiferin treated groups and “c” indicates the significant difference between the STZ+Mangiferin group and normal control group (P^a^<0.05, P^b^<0.05, P^c^<0.05).

STZ-induced diabetic rats exhibited significant alterations in the markers of nephrotoxicity. Plasma BUN, plasma creatinine, plasma uric acid and urinary albumin showed significant elevation (Figure 2A, B, C, D). However, mangiferin treatment efficiently reduced the alterations of these parameters and appeared to act as a nephroprotective agent in diabetes.

### Mangiferin protects from STZ-induced renal injury

**Figure 3:**
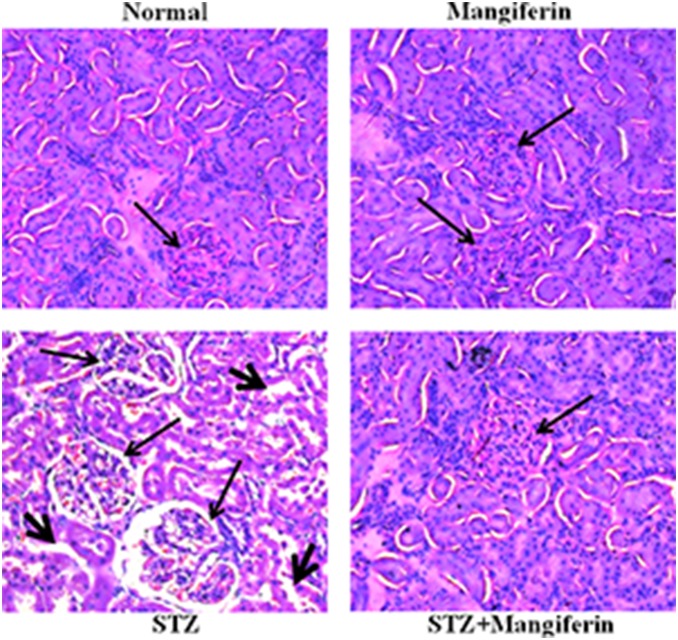
Effects of mangiferin on STZ-induced diabetic nephropathy in rats. Histological examination (H&E stained) (200x) in rat kidney of normal group, Mangiferin (Only) group, STZ-induced diabetic group, and STZ + Mangiferin group. Large arrows represent the size of glomerulus and small arrows represent the hydropic changes of the proximal convoluted tubules.

Histological studies (H&E stained) on STZ-induced diabetic kidney showed increased glomerular size and significant hydropic changes in the proximal convoluted tubules (Figure 3). These alterations were effectively decreased on post treatment with mangiferin for 30 days. These results again suggest the protective action of mangiferin in diabetic renal injury.

### Effects of mangiferin on STZ-induced oxidative stress related parameters

**Figure 4:**
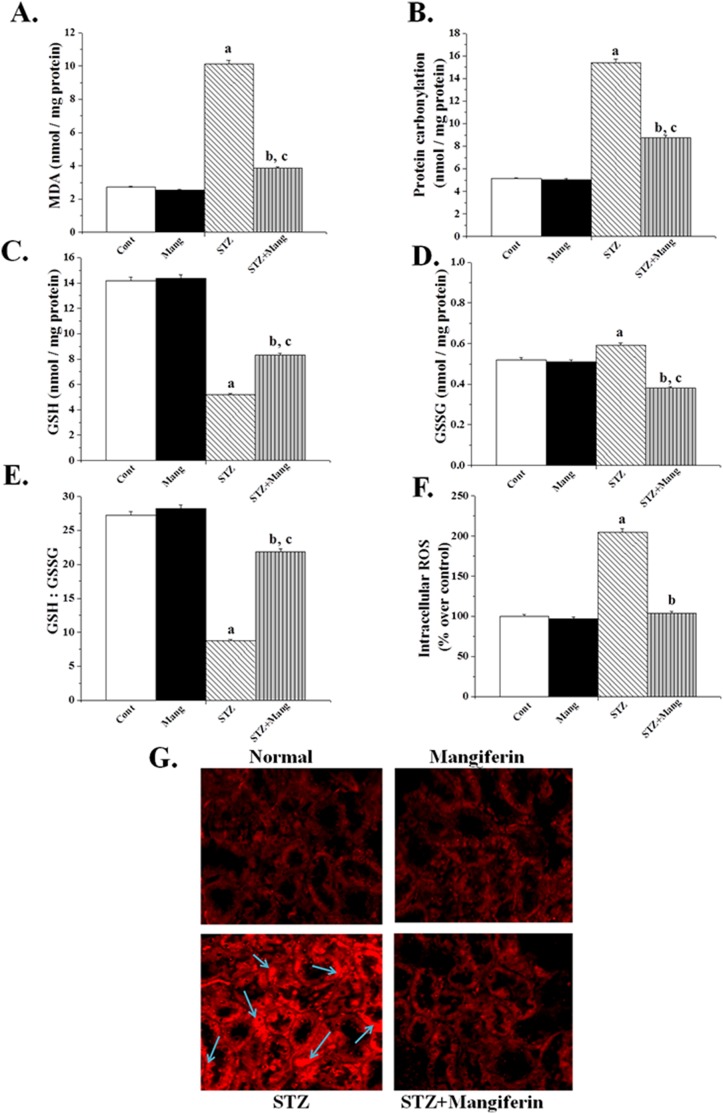
Effects of mangiferin (Mang) on STZ-induced oxidative stress related parameters and ROS levels. (A) MDA, (B) Protein carbonylation, (C) GSH (reduced glutathione), (D) GSSG (oxidized glutathione), (E) GSH to GSSG ratio, (F) Effect of mangiferin on STZ-induced intracellular ROS production in kidney and (G) Effect of mangiferin on superoxide (O_2_^.**−**^) production in kidney tissue of STZ-induce diabetic rats. Superoxide was detected by dihydroethidium (DHE) staining. Each column represents mean ± SEM, n = 6. “a” indicates the significant difference between the normal control and STZ-induced groups, “b” indicates the significant difference between the STZ-induced and mangiferin treated groups and “c” indicates the significant difference between the STZ+Mangiferin group and normal control group (P^a^<0.05, P^b^<0.05, P^c^<0.05).

In our present studies, induction of diabetes results in increased lipid peroxidation, protein carbonylation and oxidized glutathione (GSSG) content in association with decreased reduced glutathione (GSH) as well as GSH to GSSG ratio in kidney the kidney tissue (Figure 4A, B, C, D, E). However, post-treatment with mangiferin for 30 days effectively decreased the alterations in these oxidative stress related parameters suggesting it to be a good antioxidant agent that protects rat kidney from diabetes-induced oxidative damage.

### Effects of mangiferin on STZ -induced ROS levels

ROS mediated oxidative stress due to hyperglycemia plays an important role in diabetic nephropathy. In the present study, STZ-induced diabetic animals showed increased production of intracellular ROS in the kidney tissue (Figure 4F). Mangiferin, on the other hand, reduced the ROS production nearly normal level in the kidney tissue. Therefore, mangiferin in this situation acts as a powerful ROS scavenger.

We have also performed dihydroethidium (DHE) staining to assess superoxide (O_2_^.−^) production in the kidney tissue. Figure 4G clearly showed significant increase in superoxide production in the STZ-induced diabetic kidneys and its reduction by mangiferin treatment.

### Effects of mangiferin on STZ-induced changes in cellular antioxidant enzymes

**Figure 5:**
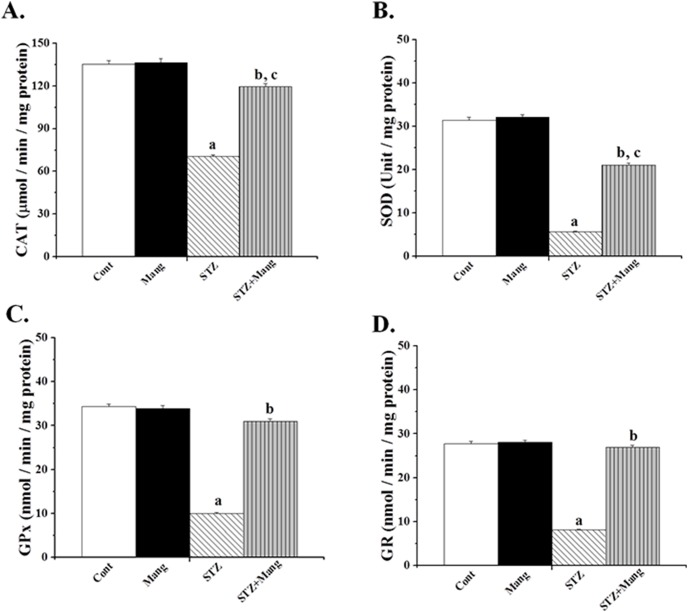
Effects of mangiferin on STZ-induced changes in cellular antioxidant enzymes. (A) CAT (Catalase), (B) SOD (Superoxide dismutase), (C) GPx (glutathione peroxidase), (D) GR (glutathione reductase). Each column represents mean ± SEM, n = 6. “a” indicates the significant difference between the normal control and STZ-induced groups, “b” indicates the significant difference between the STZ-induced and mangiferin treated groups and “c” indicates the significant difference between the STZ+Mangiferin group and normal control group (P^a^<0.05, P^b^<0.05, P^c^<0.05).

Antioxidant enzymes act as the first line of cellular defense molecules in ROS mediated oxidative damage. Effect of mangiferin on the activities of the antioxidant enzymes (CAT, SOD, GP_X_ and GR) in STZ-induced kidney was shown in Figure 5 (Figure 5A, B, C, D). In the STZ-induced diabetic kidney, the activities of these antioxidant enzymes are significantly lower. However, treatment with mangiferin for 30 days restored the activities of these antioxidant enzymes in STZ-induced diabetic kidney.

### Effects of mangiferin on STZ-induced AGE and xanthine oxidase levels

**Figure 6:**
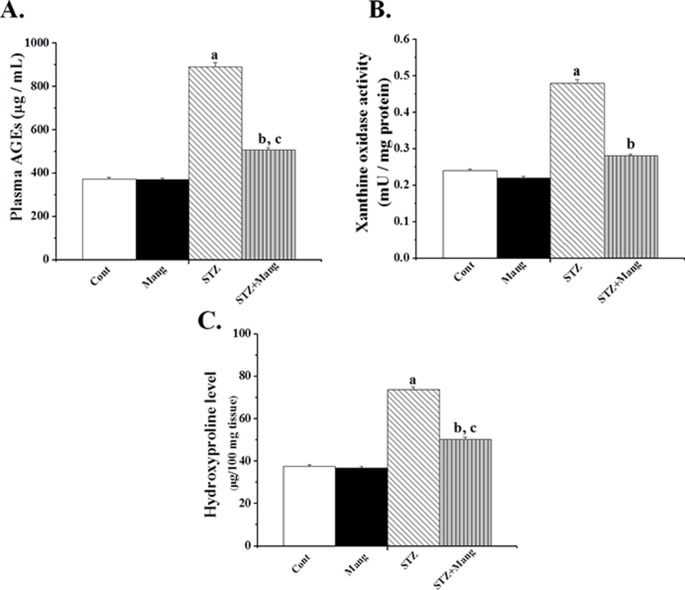
Effects of mangiferin on STZ-induced AGE, xanthine oxidase and renal hydroxyproline levels. (A) AGE (advance glycation end product), (B) xanthine oxidase and (C) renal hydroxyproline were measured. Each column represents mean ± SEM, n = 6. “a” indicates the significant difference between the normal control and STZ-induced groups, “b” indicates the significant difference between the STZ-induced and mangiferin treated groups and “c” indicates the significant difference between the STZ+Mangiferin group and normal control group (P^a^<0.05, P^b^<0.05, P^c^<0.05).

Advance glycation end product (AGE) and xanthine oxidase are important ROS inducer under diabetic condition. In the present studies, STZ-induced diabetic rats increased the plasma AGE formation and increased the activities of xanthon oxidase (in kidney tissue) (Figure 6A, B). Treatment with mangiferin for 30 days, however, suppressed AGE formation and inhibited the activities of xanthon oxidase suggesting that it could block the activities of these ROS inducers in diabetic condition.

### Effects of mangiferin on STZ-induced renal hydroxyproline levels

Collagen is normally considered as a reliable marker of fibrosis and is frequently determined by measuring the hydroxyproline content in the sample. The renal hydroxyproline level in STZ-induced diabetic rats was significantly higher (Figure 6C). However, post treatment with mangiferin in STZ-induce diabetic rats effectively reduced that hydroxyproline level. The results suggest that mangiferin could efficiently prevent renal fibrosis in diabetic rats.

### Mangiferin inhibits STZ-induced activation of PKCs

**Figure 7:**
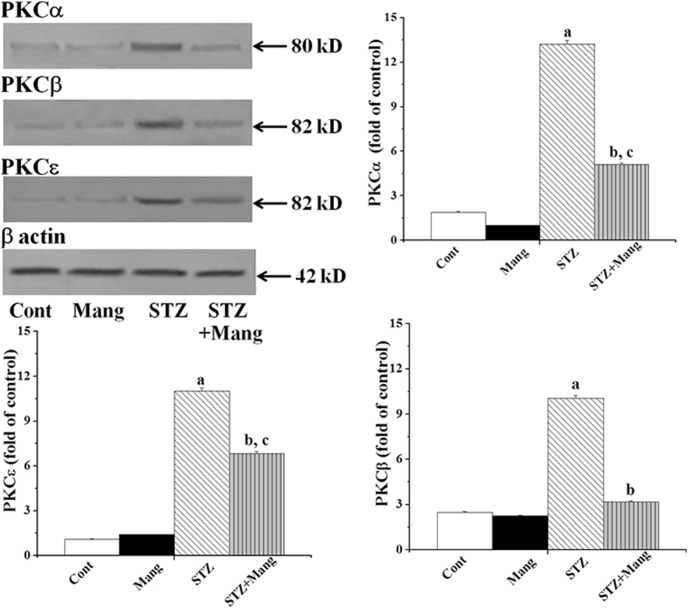
Mangiferin inhibits STZ-induced activation of PKCs (Immunoblot analysis). β actin was used as an internal control. Cont: normal control, Mang: treated with mangiferin, STZ: STZ-induced (diabetic), STZ+Mang: Mangiferin treated post to STZ-induced. PKCs (PKCα, PKCβ and PKCε expressions. Each column represents mean ± SEM, n = 6. “a” indicates the significant difference between the normal control and STZ-induced groups, “b” indicates the significant difference between the STZ-induced and mangiferin treated groups and “c” indicates the significant difference between the STZ+Mangiferin group and normal control group (P^a^<0.05, P^b^<0.05, P^c^<0.05).

Hyperglycemia induced ROS production initiates the activation of PKCs in the development of diabetic nephropathy. Immunoblot analysis showed that STZ induced diabetes was strongly associated with the increased expression of PKCα, PKCβ and PKCε in kidney tissue (Figure 7) and this expression could significantly be reduced by the treatment with mangiferin for 30 days.

### Mangiferin inhibits STZ-induced activation of MAPKs

**Figure 8:**
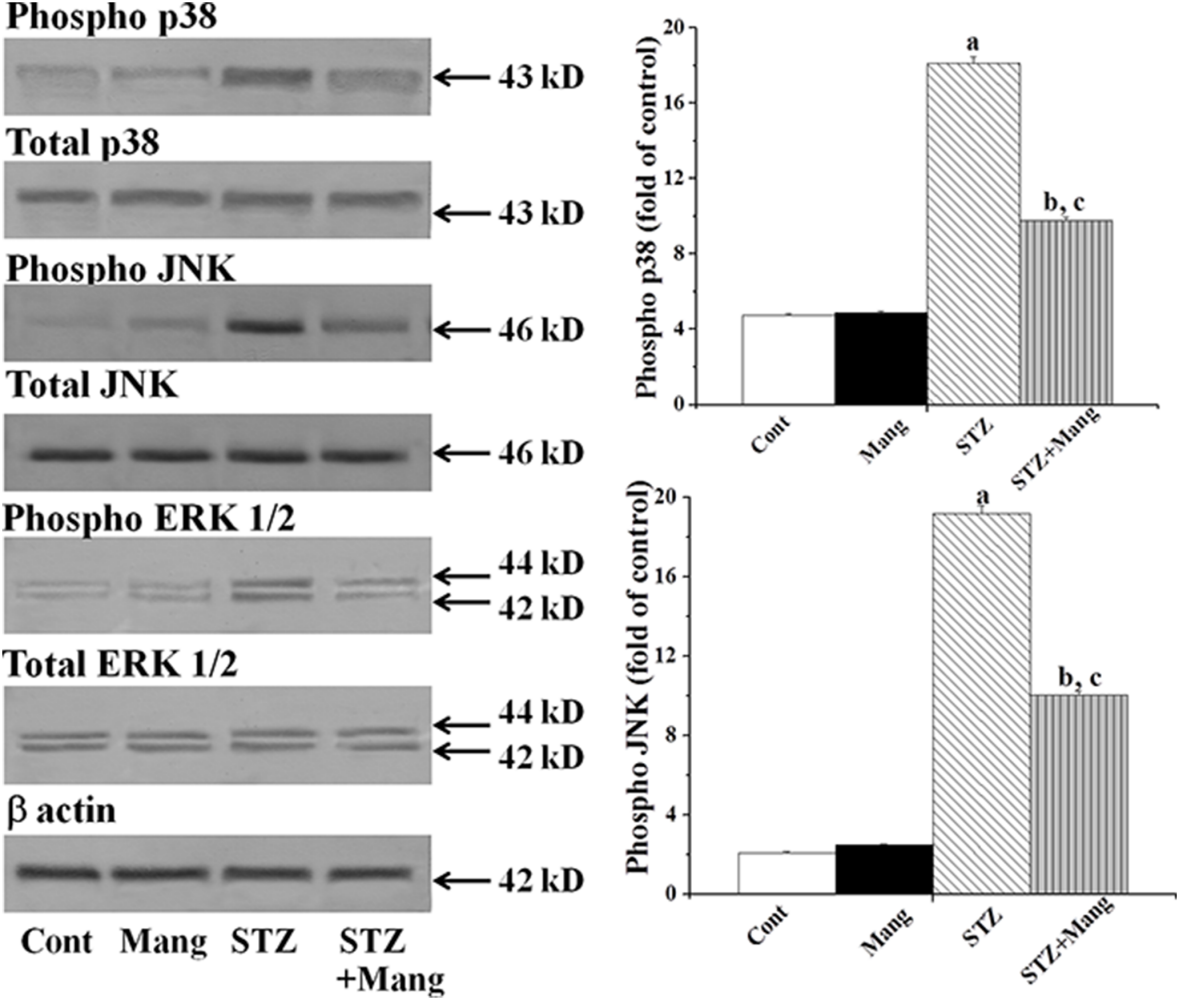
Mangiferin inhibits STZ-induced activation of MAPKs (Immunoblot analysis). β actin was used as an internal control. Cont: normal control, Mang: treated with mangiferin, STZ: STZ-induced (diabetic), STZ+Mang: Mangiferin treated post to STZ-induced. MAPKs (p38, JNK and ERK1/2) expressions. Each column represents mean ± SEM, n = 6. “a” indicates the significant difference between the normal control and STZ-induced groups, “b” indicates the significant difference between the STZ-induced and mangiferin treated groups and “c” indicates the significant difference between the STZ+Mangiferin group and normal control group (P^a^<0.05, P^b^<0.05, P^c^<0.05).

Hyperglycemia mediated oxidative stress is related to the activation of MAPKs family proteins. This family is known to act as the inducers of apoptotic cell death under a variety of pathophysiological circumstances [50]. In our present study, immunoblot analysis shows the stimulated phosphorylation of p38, JNK and ERK1/2 MAPKs in the renal tissue of STZ-induced diabetic rat (Figure 8). On the other hand, mangiferin treatment, post to diabetic induction, significantly reversed the activation of p38, JNK and ERK1/2 MAPKs.

### Mangiferin inhibits STZ-induced activation of NF-κB pathway

**Figure 9:**
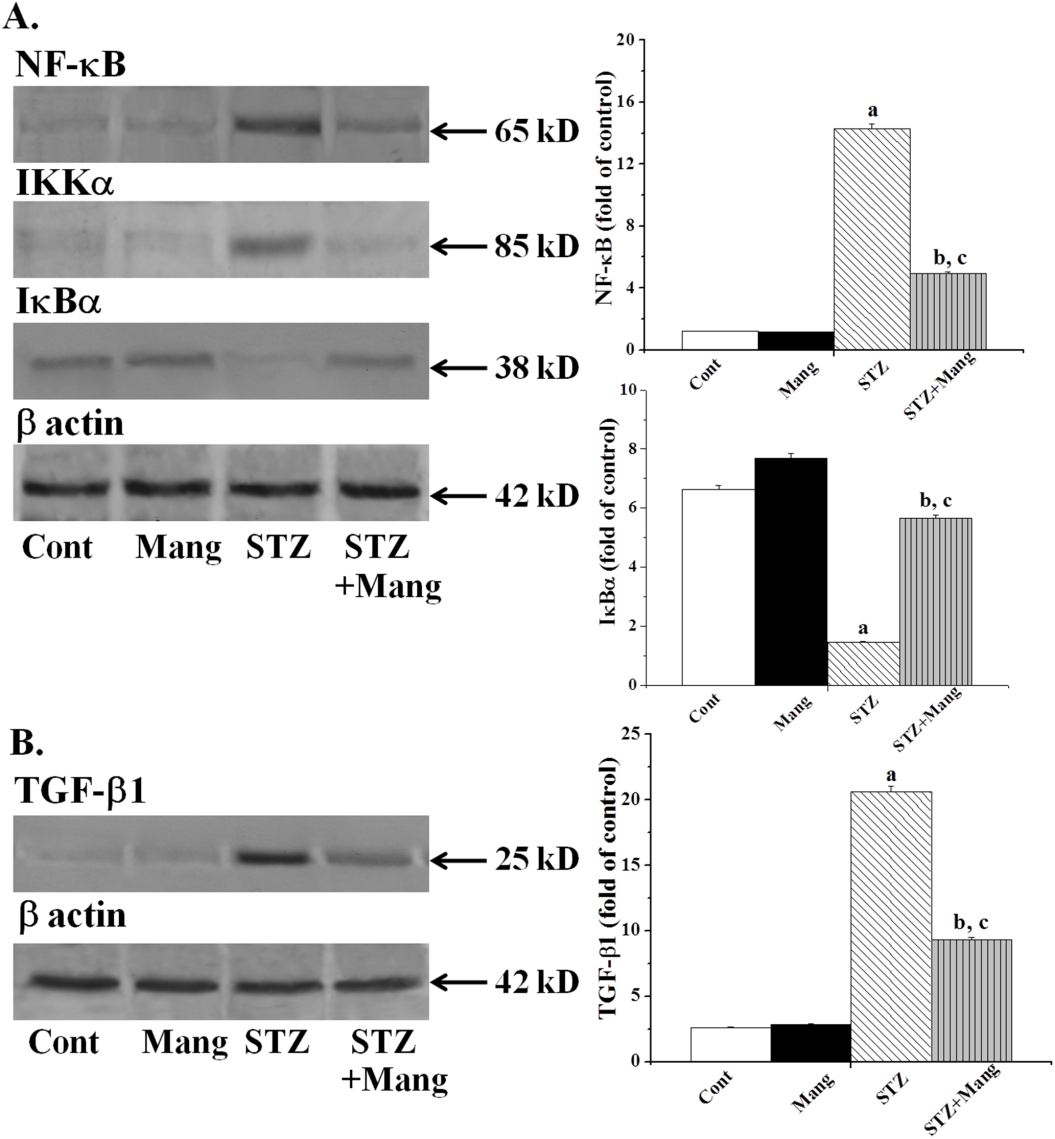
Mangiferin (Mang) inhibits STZ-induced activation of NF-κB pathway and overexpression of TGF-β1 (Immunoblot analysis). (A) NF-κB (NF-κB, IKKα and IκBα expressions and (B) TGF-β1 expressions. Each column represents mean ± SEM, n = 6. “a” indicates the significant difference between the normal control and STZ-induced groups, “b” indicates the significant difference between the STZ-induced and mangiferin treated groups and “c” indicates the significant difference between the STZ+Mangiferin group and normal control group (P^a^<0.05, P^b^<0.05, P^c^<0.05).

NF-κB, an important inflammation transcription factor, plays a critical role for the pathogenesis of diabetic nephropathy. To examine whether the activation of NF-κB pathway has any role in STZ-induced diabetic nephropathy and whether mangiferin can inhibit this occurrence, we performed an immunoblot analysis. Our result shows that, in STZ-induced diabetic kidney tissue, expression of NF-κB and the phosphorylation of IKKα increased and expression of IκBα decreased (Figure 9A). Mangiferin treatment could, however, effectively decrease the expression of NF-κB and IKKα but increased the expression IκBα. So, we say that mangiferin could inhibit diabetes induced NF-κB pathway.

### Mangiferin inhibits STZ-induced overexpression of TGF-β1

Transforming growth factor-bita 1 (TGF-β1) plays a critical role in the development of diabetic nephropathy. Immunoblot analysis shows that the expression of TGF-β1 was significantly increased in the in kidney tissue of STZ-induced diabetic rats and was efficiently inhibited by the post treatment with mangiferin (Figure 9B).

### Effects of mangiferin on STZ-induced TNFα releases

**Figure 10:**
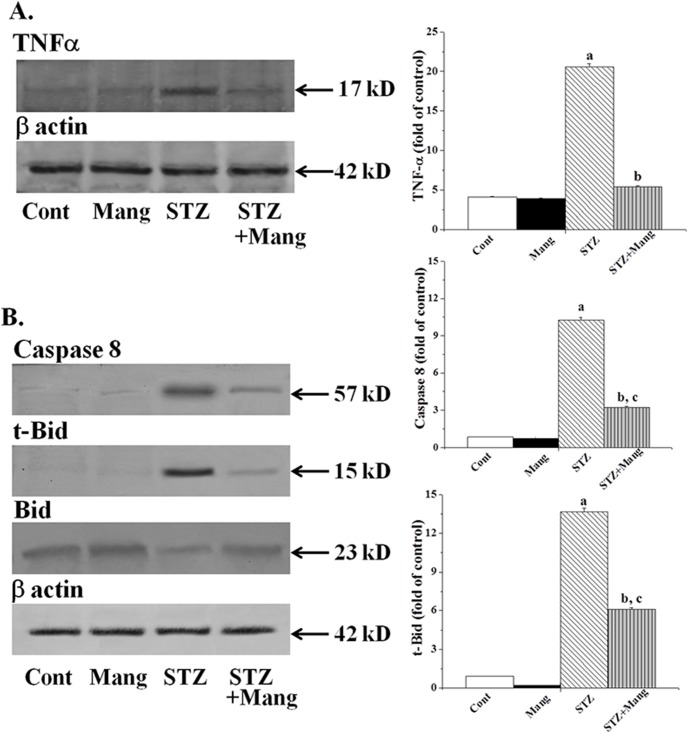
Mangiferin (Mang) inhibits STZ-induced TNFα releases and the activation of caspase 8 and t-Bid (Immunoblot analysis). (A) TNFα expression levels and (B) caspase 8, t-Bid and Bid expressions. Each column represents mean ± SEM, n = 6. “a” indicates the significant difference between the normal control and STZ-induced groups, “b” indicates the significant difference between the STZ-induced and mangiferin treated groups and “c” indicates the significant difference between the STZ+Mangiferin group and normal control group (P^a^<0.05, P^b^<0.05, P^c^<0.05).

In the present study, we have investigated the effect of mangiferin on the expression of TNFα in kidney tissues of normal and experimental rats. The expressions of TNFα were increased significantly in STZ-induced diabetic rats (Figure 10A). On the other hand, treatment with mangiferin effectively normalized the expression of TNFα. So mangiferin could successfully inhibit the TNFα in diabetic kidney.

### Mangiferin inhibits STZ-induced activation of caspase 8 and t-Bid

Caspase-8 can function independently by initiating TNFα related apoptosis in various pathophysiological conditions. Therefore, to investigate whether the activation of TNFα related caspase 8 and t-Bid (cleaved form of Bid) has any role in STZ-induced diabetic nephropathy and whether mangiferin can inhibit these activations; immunoblot analysis has been carried out using appropriate antibodies. Our results reveal that the expression of caspase 8 and t-Bid was significantly increased although the expression of Bid in STZ-induced diabetic kidney tissue of experimental rats was decreased. Mangiferin post-treatment effectively reversed these expressions (Figure 10B). So these results suggest that mangiferin could efficiently suppress the TNFα related apoptotic pathway.

### Anti-apoptotic effects of mangiferin on STZ-induced Bcl-2 family proteins

**Figure 11:**
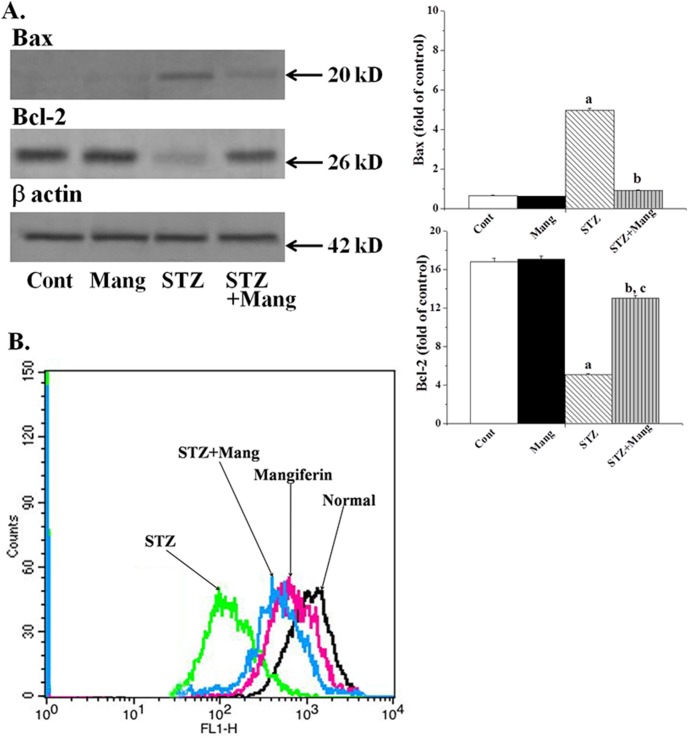
Mangiferin ameliorates Bcl-2 family proteins (Immunoblot analysis) and inhibits the mitochondrial dependent apoptotic pathways in STZ-induced diabetic kidney of rats (Immunoblot analysis). (A) Expressions of pro-apoptotic (Bax) and anti-apoptotic (Bcl-2) Bcl-2 family proteins, (B) mitochondrial membrane potential (flow cytometry). Each column represents mean ± SEM, n = 6. “a” indicates the significant difference between the normal control and STZ-induced groups, “b” indicates the significant difference between the STZ-induced and mangiferin treated groups and “c” indicates the significant difference between the STZ+Mangiferin group and normal control group (P^a^<0.05, P^b^<0.05, P^c^<0.05).

In hyperglycemia mediated oxidative stress, proapoptotic (Bax, Bad etc.) and antiapoptotic (Bcl-2, Bcl-xl) Bcl-2 family proteins are involved in response to apoptosis. Immunoblot analysis shows that the expression of Bcl-2 (antiapoptotic) was downregulated and that of Bax (proapoptotic) was upregulated in STZ-induced diabetic renal tissue (Figure 11A). However, treatment with mangiferin significantly altered the expression of these proteins. So, mangiferin could act as an effective anti-apoptotic agent by increasing the expression of anti-apoptotic Bcl-2 family proteins in the mitochondria of diabetic kidney.

### Anti-apoptotic effects of mangiferin on STZ-mediated mitochondrial dependent apoptotic pathways

**Figure 12:**
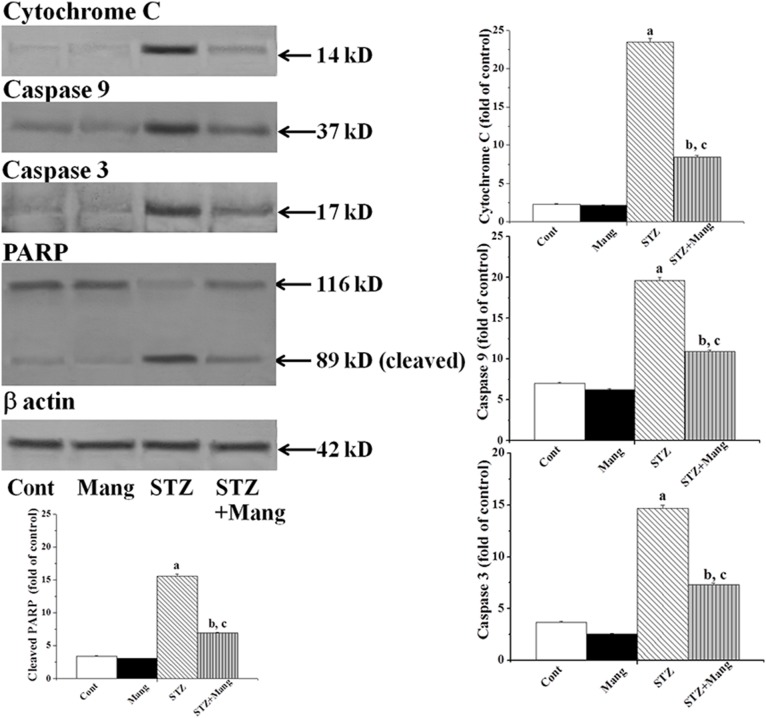
Mangiferin inhibits the mitochondrial dependent apoptotic pathways in STZ-induced diabetic kidney of rats (Immunoblot analysis). Cytochrome C, Caspase 9, Caspase 3 and PARP expressions. Each column represents mean ± SEM, n = 6. “a” indicates the significant difference between the normal control and STZ-induced groups, “b” indicates the significant difference between the STZ-induced and mangiferin treated groups and “c” indicates the significant difference between the STZ+Mangiferin group and normal control group (P^a^<0.05, P^b^<0.05, P^c^<0.05).

Translocation of Bax (pro-apoptotic Bcl-2 family protein) from cytosol to mitochondria in hyperglycemia induces oxidative stress and causes damages to the outer mitochondrial membrane; which in turn, initiates the mitochondrial dependent apoptotic pathway [51]. To establish whether mangiferin applies its anti-apoptotic activities in the STZ-induced mitochondrial dependent apoptotic pathway, we measured the mitochondrial membrane potential in the kidney tissue (by flow cytometry) as well as the expression cytosolic cytochrome C, caspase 9, caspase 3 and PARP cleavage in kidney tissue (by immunoblot analysis). Results showed that STZ-induced diabetic condition significantly reduced the mitochondrial membrane potential (MMP) (Figure 11B), increased the expression of cytosolic cytochrome C, caspase 3 as well as caspase 9 and PARP cleavage (Figure 12). However, treatment with mangiferin, post to STZ exposure, effectively inhibited these parameters suggesting that mangiferin has a potential anti-apoptotic effect in diabetes-mediated mitochondrial dependent apoptotic pathways in kidney.

### Mangiferin protects from STZ-induced apoptosis in kidney

**Figure 13:**
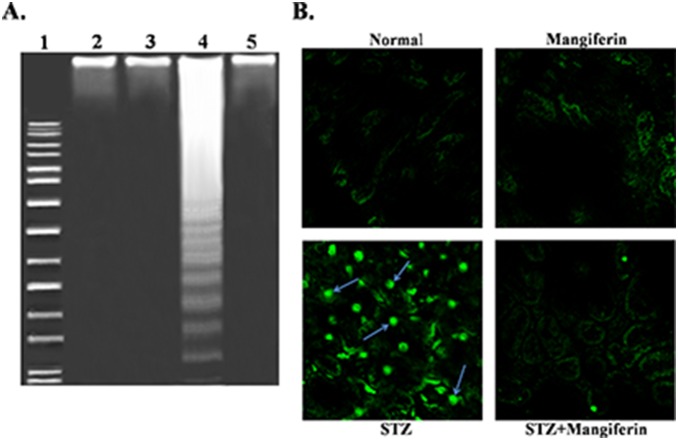
Mangiferin protects from STZ-induced apoptosis in kidney. (A) DNA fragmentation on agarose/ethydium bromide gel. DNA isolated from experimental rat kidney was loaded onto 1% (w/v) agarose gels. Lane 1: Marker (1 kb DNA ladder); Lane 2: DNA isolated from normal kidney tissue; Lane 3: DNA isolated from mangiferin treated kidney tissue; Lane 4: DNA isolated from STZ-induced diabetic kidney; Lane 5: DNA isolated from Mangiferin treated in STZ-induced diabetic kidney. DNA ladder formation in STZ-induced diabetic kidney. (B) TUNEL staining in kidney tissue sections (10x) in experimental rats. Arrows indicate TUNEL positive nucleus.

STZ-induced apoptosis has also been evident from the ladder pattern (hallmark of apoptosis) in DNA gel electrophoresis (Figure 13A) and TUNEL assay (Figure 13B). In the figure, TUNEL positive nuclear staining has been shown in STZ-induced diabetic rat kidney representing the apoptosis of kidney cells in this pathophysiology. On the other hand, mangiferin significantly reduced the STZ-induced disturbances in the number of TUNEL positive nuclei and protected DNA in the tissue. Data from the DNA fragmentation assay and the TUNEL assay confirm the anti-apoptotic role of mangiferin in diabetic nephropathy.

## Discussion

**Figure 14:**
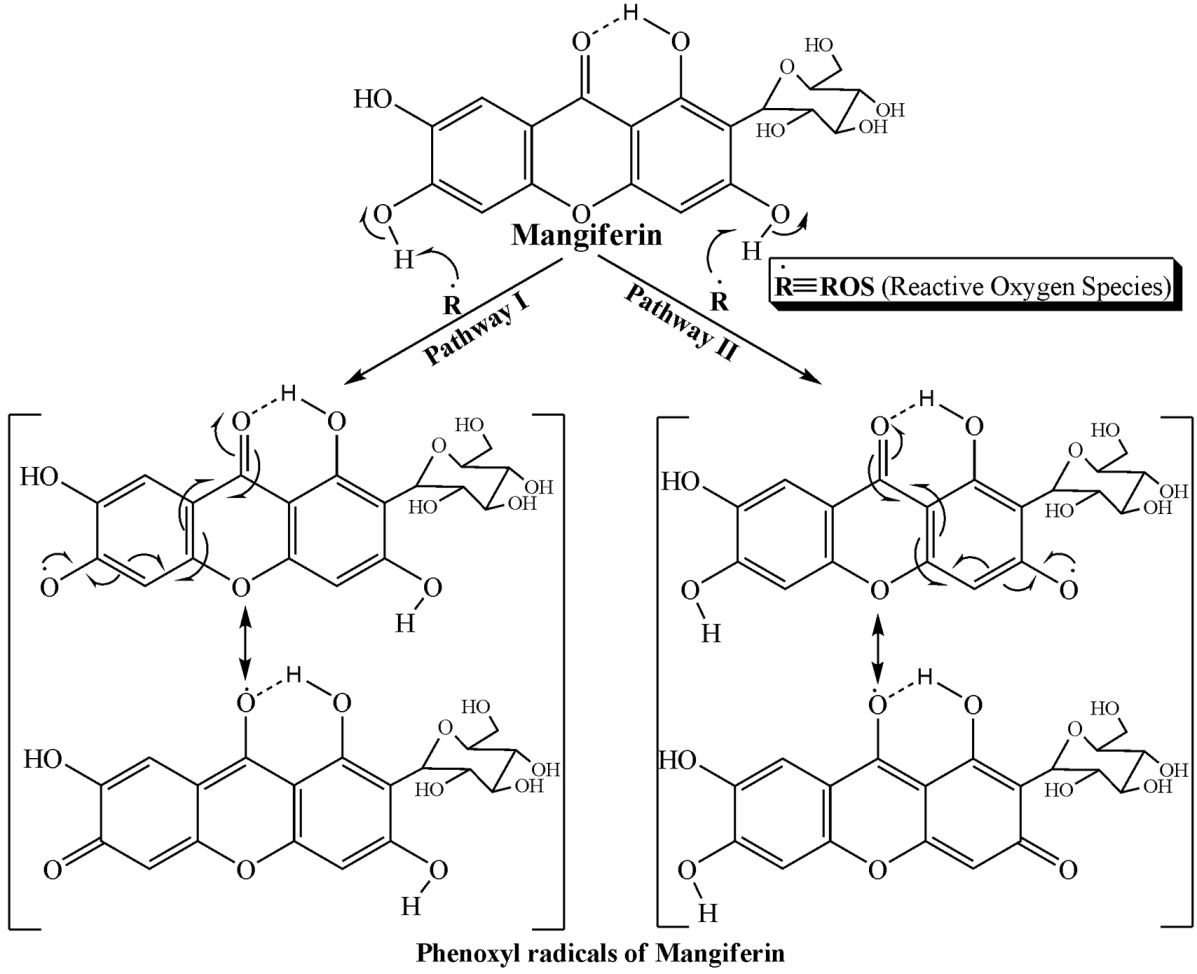
Plausible molecular mechanisms of mangiferin how it scavenge reactive free radicals (ROS).

Various experimental and clinical reports suggest that oxidative stress plays a major role in the pathogenesis of diabetic nephropathy in both type 1 and type 2 diabetes mellitus [52], [53]. Our present study established that mangiferin could provide protection against diabetic nephropathy (STZ; 65 mg/kg b.w. single dose) [54] via the reversal of the activation of PKCs, MAPKs, NF-κB as well as TGF-β1 and inhibiting both the extrinsic (mitochondrial independent) and intrinsic (mitochondrial dependent) apoptotic cell death involved in this pathophysiology. A number of recent studies showed that mangiferin could protect various organs including the kidney, probably because of its strong anti hyperglycemic effect and free radical (ROS) scavenging activity [55]–[57] in STZ-induce diabetic condition. The free radical scavenging property of mangiferin is probably understood from the structural point of view as it contains four phenolic H-atoms, two of which could easily be abstracted by suitable free radicals (e.g., ROS) to form two phenoxyl radicals that are stabilized by resonance (Figure 14) [58], [36]. Furthermore, mangiferin could decrease the xanthon oxidase activation and advance glycation end product (AGE) formation; these two phenomena are considered to be the crucial mechanism for increased production of ROS. In this study, we characterized diabetes mellitus by increased plasma glucose level along with decreased kidney to body weight ratio. A significant increase in plasma BUN, creatinine, uric acid and urinary albumin also indicated the progressive nephrotoxicity in the animals. Mangiferin, on the other hand, effectively reversed this pathophysiology by lowering plasma BUN, creatinine, uric acid and urinary albumin. Mangiferin also reduced the plasma glucose level, restored kidney to body weight ratio and altered diabetes-induced oxidative stress related parameters such as MDA content, protein carbonylation, ROS production, GSH and GSSG levels as well as GSH:GSSG ratio in the kidney tissue.

Besides dietary antioxidants, cells also develop numerous antioxidant defense systems against free radical assaults. GSH plays a major role in protecting cells against oxidative stress and it is decreased in the kidney due to STZ-mediated oxidative stress. GR reduces GSSG to GSH, thereby supporting the antioxidant defense system. GR has a disulfide bond in its active site, but ROS interferes with the disulfide bond and inhibits the enzyme. SOD and CAT mutually plays a vital role in keeping out ROS. GPx requires GSH in their course of reactions for removal of excess free radicals from the system. Decreased GSH content occurs because of the reduced functions of GPx, making cells more susceptible to oxidative damage. In the present study, we found that the mangiferin effectively attenuated the decreased activities of antioxidant enzymes such as CAT, SOD, GP_X_ and GR in STZ-induced diabetic kidney.

Kidney tubule fibrosis plays an important role in the development of diabetic nephropathy [59]. In our present study, we observed a significant increase in the levels of renal hydroxyproline content in STZ-induce diabetic rats; this enhancement in turn increases the severity of the kidney lesions and fibrosis in experimental animals. Mangiferin, on the other hand, effectively diminished the hydroxyproline level, suggesting its anti-fibrotic efficacy in diabetic condition.

Hyperglycemia contributes to the formation of AGEs in diabetic condition and AGEs has been considered as an important biological source of ROS. Increased amounts of AGEs have been reported to be present in diabetic renal glomeruli [60]. AGEs can form from intracellular auto-oxidation of glucose to glyoxal (activation of polyol pathway also occur in hyperglycemic condition), breakdown of the Amadori product (1-amino-1-deoxyfructose lysine adducts) to 3-deoxyglucosone and fragmentation of glyceraldehyde-3-phosphate and dihydroxyacetone phosphate to methylglyoxal. These reactive intracellular dicarbonyls (such as glyoxal, methylglyoxal and 3-deoxyglucosone) react with amino groups of intracellular and extracellular proteins to form AGEs [8]. STZ also considerably increased the activity of xanthine oxidase considerably, which is another important biological source of ROS in diabetic conditions [3]. However, our present study showed that mangiferin could suppress the AGEs formation and inhibit the activities of xanthon oxidase in STZ-induced diabetic renal tissue.

Investigation of the ROS activated signal transduction pathways in STZ-induced diabetic renal dysfunction showed that ROS could activate PKCs, MAPKs and transcription factor (NF-κB); and could also upregulate TGF-β1. Various reports suggest that hyperglycemia mediated and ROS-induced activation of PKCs and MAPKs play a significant role in the development and progress of diabetic nephropathy [61], [62]. Lee et al. (2003) [61] also reported that the activation PKCs is also responsible for the generation of ROS in STZ-induced diabetic kidney. In our present study, we observed the increased expression of PKCs (PKCα, PKCβ and PKCε) and MAPKs (phospho- p38, phospho-JNK and phospho-ERK1/2) in STZ-induced diabetic kidney. However, treatment with mangiferin, inhibits the activations of ROS-mediated PKCs as well as MAPKs suggesting that STZ-induced diabetic nephropathy is mediated via the activation of PKCs MAPKs family proteins. A previous report suggests that hyperglycemia mediated ROS induces the activation of the transcription factor, NF-κB [63]. Due to the activation of MAPKs family proteins in our present study in STZ-induced diabetic kidney, the transcription factor, NF-κB, was also activated. Activation of this transcription factor could be regulated by the phosphorylation of its p65 subunits and degradation of its inhibitor-κB (IκB) via phosphorylation of IKKα/β resulting its translocation into the nucleus [64]. Then, transcription of DNA to mRNA occurs followed by its translation into protein, helping cellular apoptosis. NF-κB also up-regulates TGF-β1 [65] which plays an important role in the development of renal hypertrophy and the accumulation of extracellular matrix (ECM) resulting in the development of diabetic nephropathy in experimental animals [66], [67]. In our present study we also observed that STZ up-regulated the TGF-β1 in the kidney. Mangiferin, could, however, inhibit the activation of NF-κB and attenuate the up-regulated TGF-β1 in this pathophysiological condition. Finally we suggest that mangiferin could inhibit hyperglycemia-mediated and ROS-induced activation of signal transduction cascade such as PKCs, MAPKs and transcription factor (NF-κB) as well as up-regulation of TGF-β1 in diabetic kidney.

Our recent interest has also been focused to determine the role of the most important cytokine, TNFα, released in the inflammatory process which can activate signaling pathways related to cell survival and apoptosis (extrinsic apoptosis) in diabetic circumstances. TNFα initiates the activation of caspase 8 in the cytosol via its binding to the death receptor, TNF-R1. Caspase 8, in this scenario, plays the most important role in the implementation of programmed cell death [68] via two different pathways, either type I or type II. In type I pathway, the initiator caspase, caspase-8, directly activates the downstream effector caspase, caspase 3 is subsequently cleaved resulting in apoptosis [69], [70]. In type II pathway, caspase 8 does not directly activate the caspase 3 to execute cell death. In this process, caspase 8 executes cell death via mitochondria-dependent apoptotic pathways and is going through mitochondrial Bcl-2 family protein, Bid. Bid is cleaved to 15 kD truncated form (t-Bid) by caspase-8 which in turn translocates to the mitochondria and promotes mitochondria-dependent apoptotic pathways [71]. In the present study, we found that STZ-induced diabetic kidney could release TNFα which in turn activates caspase 8 followed by the activation of mitochondrial dependent cell death pathways (type II) via the cleavage of Bid to t-Bid in the cytosol. Mangiferin, on the other hand, inhibits these TNFα mediated apoptotic events via decreased level of released TNFα as well as decreased expression of caspase 8, t-Bid and increased expression of Bid in the cytosol.

Bcl-2 family proteins are the family of proteins involved in programmed cell death due to oxidative stress and mitochondria and play a vital role in the regulation of this process. ROS mediated oxidative stress disturbs the balance between pro-apoptotic (such as bcl-2 and bcl-XL) and anti-apoptotic (such as Bad, Bax or Bid) Bcl-2 family proteins, resulting an excess of pro-apoptotic proteins in the cells which are more susceptible to apoptosis. An excess of pro-apoptotic Bcl-2 proteins at the surface of the mitochondria is considered to be an important factor for the development of the Permeability Transition (PT) pore due to the loss of mitochondrial membrane potential and released cytochrome C in cytosol. Cytochrome C efficiently to interacts with Apaf-1 and leads to the recruitment of pro-caspase 9 to form a multiple-protein complex called the apoptosome. Apoptosome formation leads to the activation of caspase 9 as well as caspase 3. Caspase 3 plays a very important role in executing apoptosis by activating DNases and inhibiting the important DNA repair enzyme poly (ADP-ribose) polymerase (PARP). The function of PARP (repair DNA damage) is prevented by caspase 3 via the cleavage of PARP. On the other hand, the fragmentation of DNA is due to the enzyme caspase activated DNase (CAD), which exists as ICAD (inhibitor of CAD; an inactive complex) in normal condition. When caspase 3 is activated in stressed condition, ICAD is cleaved to CAD by caspase 3 as well as fragmentation of chromosomal DNA into nucleosomal units and ultimately apoptotic cell death occurs [72]. In our studies, we found the increased expression of Bax (pro-apoptotic), decreased expression of Bcl-2 (anti-apoptotic) as well as activated mitochondrial dependent pathways via reduced mitochondrial membrane potential, enhanced cytochrome C, increased expression of caspase 9 and caspase 3 in the cytosol and cleavage of PARP in STZ-induced diabetic nephropathy. However, treatment with mangiferin, effectively ameliorates the changes in Bcl-2 family proteins and inhibits the mitochondrial dependent apoptotic pathways in this pathophysiology. STZ-induced renal apoptotic cell death and the protective action of mangiferin was also confirmed from DNA fragmentation analysis (ladder formation) and TUNEL assays (showing TUNNEL positive nuclear staining).

**Figure 15:**
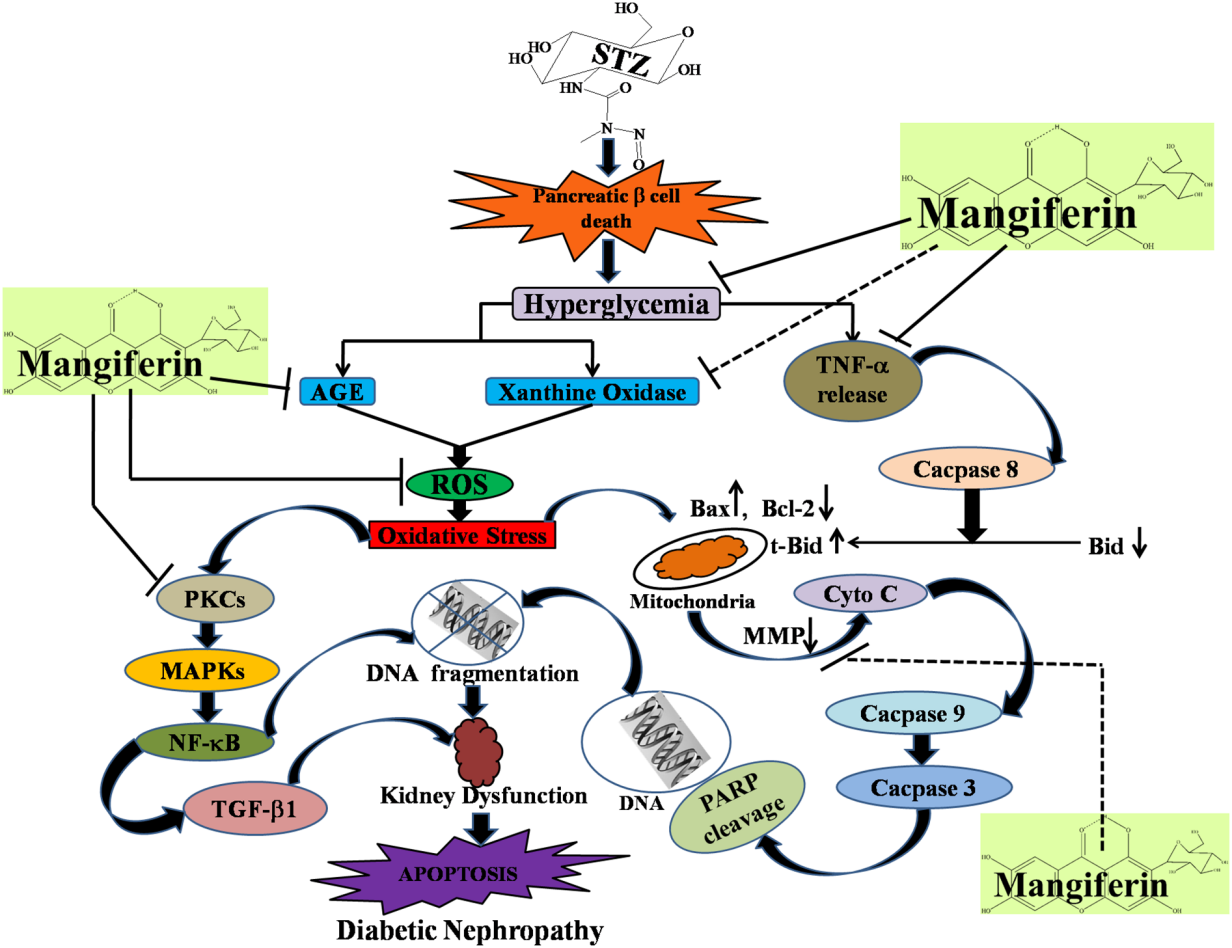
Schematic representation of Pb(II) induced hepatotoxicity and its protection by mangiferin.

In conclusion, the findings of our present study established, for the first time, that mangiferin treatment could provide effective protection against oxidative injury in the renal tissue of STZ-induced type 1 diabetic rats via ROS-induced, PKCs, MAPKs, NF-κB and TGF-β1 mediated, TNFα related mitochondrial dependent apoptotic pathways (Figure 15).
