# Correction: Mangiferin Attenuates Diabetic Nephropathy by Inhibiting Oxidative Stress Mediated Signaling Cascade, TNFα Related and Mitochondrial Dependent Apoptotic Pathways in Streptozotocin-Induced Diabetic Rats

**DOI:** 10.1371/journal.pone.0115364

**Published:** 2014-12-03

**Authors:** The PLOS ONE Staff

There is an error in the legend for Figure 15. Please see the complete, correct Figure 15 and legend here.

**Figure 15:**
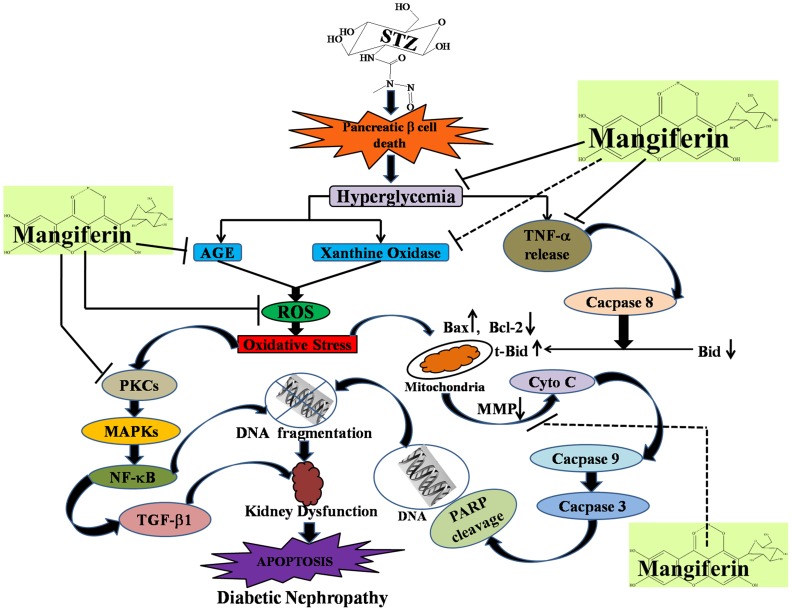
Schematic representation of STZ induced diabetic nephropathy and its protection by mangiferin.
